# Nonspecific Headache Diagnosed as a Brain Colloid Cyst with Hydrocephalus

**DOI:** 10.5811/cpcem.2016.12.32939

**Published:** 2017-03-06

**Authors:** Christine Carroll, Mark Riddle

**Affiliations:** Carl R. Darnall Army Medical Center, Department of Emergency Medicine, Fort Hood, Texas

## Abstract

A 29-year-old male presented to our emergency department with complaints of a left frontal headache, similar to his prior headaches. He also reported about 30 minutes of facial and tongue numbness, left arm weakness, slurred speech and changes in hearing that had resolved prior to his arrival. Despite the short duration of the other neurologic symptoms, he also endorsed persistent “dizziness.” Despite his history of recurrent headaches, he had never had any neuroimaging. This, as well as his reports of new neurological symptoms, prompted his care team to obtain a non-contrast brain computed tomography. The findings were consistent with a mass with mild hydrocephalus. Patient underwent neurosurgical resection of the mass at a tertiary center. He did well after surgery and was discharged on postop day 6 with a diagnosis of colloid cyst.

## INTRODUCTION

Colloid cysts (CC) are slow growing, benign developmental lesions that occur at the roof of the third cerebral ventricle close to the foramen of Monroe.^1^,^2^,^3^ They account for 0.5%–2% of all intracranial^1^,^2^,^3^ and 15%–20% of all intraventricular tumors.^1^ They may also be found in other locations such as the fourth ventricle, posterior fossa, brainstem or cerebellum.^2^ Men are more affected than women, and they are diagnosed mostly in the third and fifth decades.^3^ CCs generally range in size from 2–50mm in diameter,^1^,^3^,^4^ and they may contain colloid material including cholesterol and fats.^3^ Despite their rare occurrence, these cysts are of important clinical significance. Because of a ball-valve effect within the ventricle, they can interfere with the cerebrospinal fluid (CSF) outflow and cause hydrocephalus, irrespective of their size.^1^,^3^,^4^ Because of this effect, early detection and treatment is recommended as they can cause acute deterioration and even sudden death, which is more common if the cyst is more than 1cm in diameter.^1^,^4^

## CASE REPORT

A 29-year-old male with a past medical history only significant for hypertension arrived to our emergency department (ED) with complaints of headache and dizziness. The patient stated that in the past he had experienced similar headaches of similar intensity; however, with this headache he experienced changes in vision, which was a new phenomenon. He described his headache as frontal and stated it started about three hours prior to arrival. Shortly after the onset of the headache he had approximately 30 minutes of blurry vision, numbness to left face, tongue and left arm. His wife also noticed slurred speech with deficits in hearing that also lasted approximately 30 minutes. The neurologic symptoms completely resolved by the time he arrived to the ED; however, his headache continued and he was experiencing worsening dizziness.

**Image 1:**
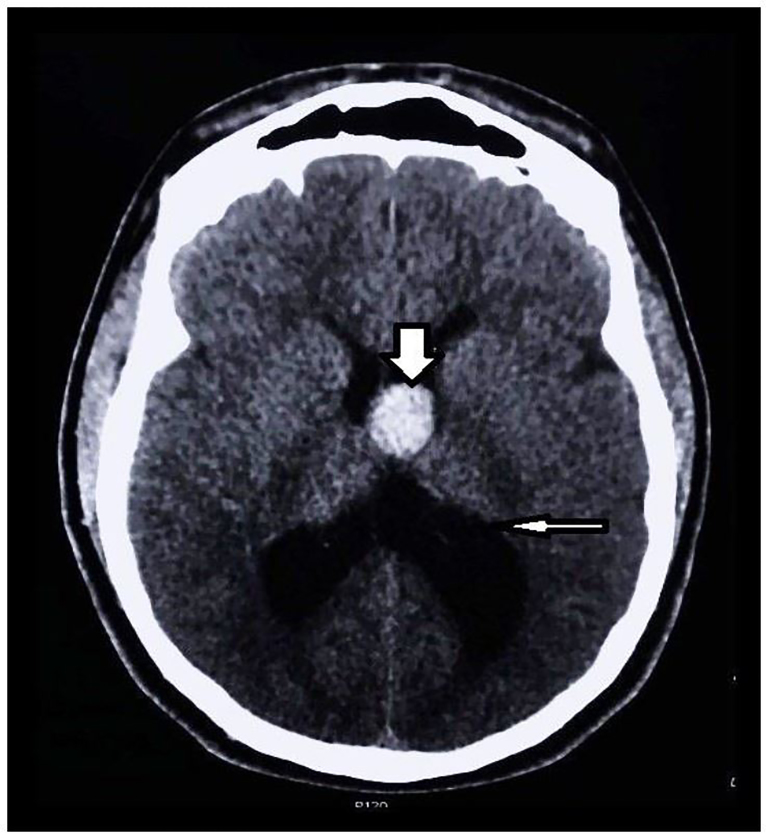
Initial noncontrast computed tomography head demonstrating the presence of the colloid cyst (thick arrow) with associated hydrocephalus (thin arrow).

Upon arrival, his blood pressure was 151/88, pulse 80, pulse ox 99% and temperature of 97.9. Blood glucose was 132. Visual acuity was normal uncorrected. Electrocardiogram (EKG) showed normal sinus rhythm with normal intervals. Lab work obtained included a complete blood count, complete metabolic panel, and troponin I, which were found to be unremarkable. The only medication the patient was taking was lisinopril (10mg) for hypertension. He had an unremarkable physical exam. He endorsed a history of similar headaches, except for his complaint of subjective dizziness, and we found no neurologic deficits on our exam. Pupils were round and reactive to light. There was no peripheral vision deficit. Speech was clear. Cranial nerves II–XII were intact. Lungs were clear to auscultation bilaterally. Cardiac and abdominal exams were benign. Extremities had 5/5 strength bilaterally, 2/4 patellar reflexes and sensation was intact. Romberg test was negative. Recurrent headaches without prior neuroimaging and his reported neurologic phenomena prior to arrival prompted a non-contrast computed tomography (CT) of his brain in the ED. The CT revealed a 2.3 cm tumor versus mass versus CC in the third ventricle causing hydrocephalus (Image 1). Because of the patient’s presentation and the CT findings the decision was made to transfer him to a tertiary center with neurosurgery service available.

At the tertiary center the patient underwent magnetic resonance imaging of the brain with contrast that revealed a 1.2×1.7×2 cm lobulated mass at the anterior aspect of the third ventricle with mild hydrocephalus without mass defect. Patient was started on levetiracetam, labetalol, hydralazine and cefazolin sodium. On hospital day 2 he had an endoscopic resection of the mass through a frontal incision and an intraventricular shunt was placed. The mass was sent for pathology, which reported results consistent with a CC.

**Image 2:**
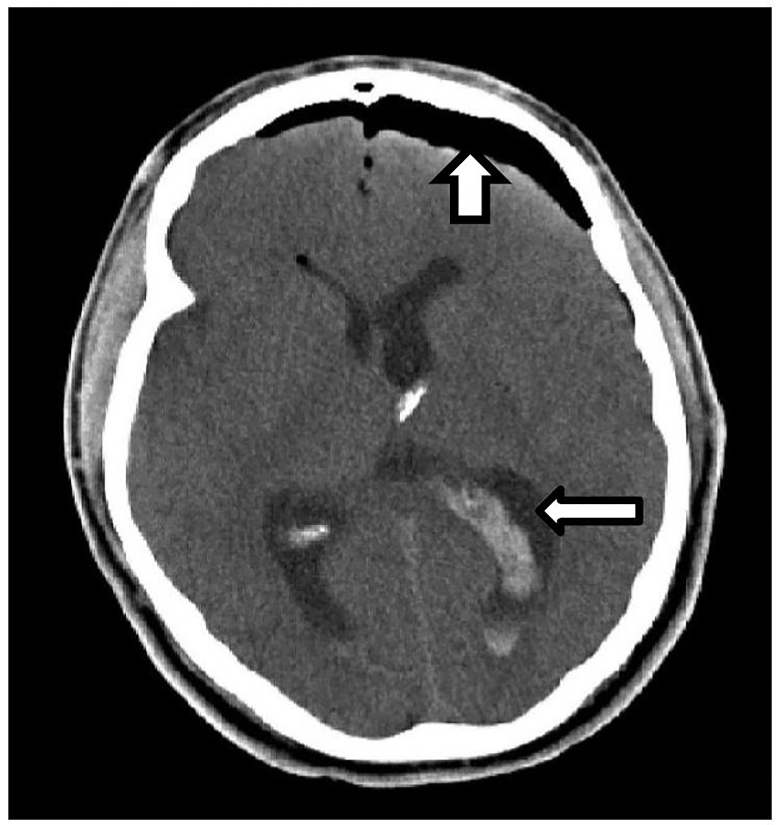
Postoperative noncontrast computed tomography head with presence of pneumocephalus (thick arrow) and intraventricular hemorrhage (thin arrow).

On postop day 1 a head CT was done secondary to patient complaining of continued headache. It demonstrated a moderate amount of intraventricular hemorrhage, pneumocephalus and subcutaneous gas seen along left prefrontal soft tissues (Image 2). All findings were thought to be secondary to surgery.

On postop day 3 the patient complained of headache with photophobia. Repeat head CT demonstrated mild residual ventriculomegaly with interval decrease in postop pneumocephalus and decrease in the intraventricular hemorrhage.

The patient continued to improve, had normal vitals and normal lab values and he was released home on postop day 6.

## DISCUSSION

More than three million people present yearly to the ED with headaches as the presenting symptom.^5^ The emergency physician (EP) has two major responsibilities in diagnosis and management of headaches. These responsibilities are to simultaneously evaluate for life-threatening causes of headaches in a timely manner and treat the patient to alleviate suffering.^5^ Following diverse algorithms of classification and management of headaches may distract the EP from the above most important priorities in ED.^5^ Anchoring on a “headache similar as those in the past” statement can lead to a delayed or missed diagnosis with catastrophic consequences for the patient. Other pitfalls that can lead to missed diagnoses are the following: relying on relief of pain with medications as an end of diagnostic evaluation, dismissing the possibility of secondary headache in patients with known primary headaches, and believing that the cause of headache is hypertension.^5^ The prudent EP would thus have a high index of suspicion for any headache that is sudden and at its maximal intensity at onset and if the patient expresses concerns that the symptoms deviate from their regular pattern. Imaging is recommended if any of the “red flags” are present: focal neurologic deficits, any cranial nerve abnormality, or onset of new symptoms with prior diagnosis of primary headaches. Any of the above presentations can be more or less obvious in patients who have an undiagnosed CC.

Headache is present in 68–100% of patients with CCs.^3^,^4^,^6^ The headaches can be intermittent or severe and are often relieved by recumbency or sleep.^3^,^4^ The headaches usually start in the frontal area and are characterized by their short duration, often improving with changes in position.^4^ Other common symptoms are nausea, vomiting, visual changes, short-term memory loss and gait disturbances generally caused by obstruction of the foramen of Monroe.^3^,^4^,^6^ Less common symptoms are stroke, psychiatric problems, incontinence, behavioral changes, generalized weakness, syncope and sudden death.^4^ In rare cases they may present with endocrine abnormalities such as oligomenorrhea, galactorrhea and hypogonadotropic hypogonadism.^6^

Patients may have frequent similar headaches that are generally responsive to pain control measures, potentially leading to a delayed diagnosis in the ED. Patients like ours may describe them as similar as prior headaches, which can decrease the EP’s concern and delay brain imaging. Despite availability of early diagnosis with imaging, other conditions must be kept in mind to reduce delays in treatment. These may include things such as CCs, arachnoid cysts, craniopharyngioma, pituitary gland tumors, aneurysms, or Rathke cleft cysts.^2^,^6^

There is no general agreement on the treatment of CCs, and the optimal management has been a debatable issue. Some of the treatment options include open and microsurgical resection, stereotactic aspiration, endoscopic removal, or simple shunt application.^1^,^4^,^6^ Some neurosurgeons suggest aggressive resection of CCs, while others recommend only fenestration and suction of the cyst contents since they believe these cysts usually do not relapse.^6^ However, despite the lack of agreement of how to best treat the CCs, the microsurgical approach has been considered the gold standard for treatment since complete resection can be achieved.^1^ Should endoscopic resection be chosen as the treatment option, it has been shown to have lower complication rates, less surgery time, shorter hospital stay and lower infection rates, but the cysts tend to recur more often than with microsurgery.^1^

## CONCLUSION

In conclusion, physicians should have a low threshold for imaging presenting headaches with subjective changes from baseline even if neurologic exam is normal. Shared decision-making and listening to the patient along with a thorough physical exam is of utmost importance. In our case, the patient’s increasing concerns regarding his symptoms along with the changes noted by his wife ultimately led us to the final unexpected diagnosis.

## References

[1] Sharifi G, Bakhtevari MH, Samadian M (2015). Endoscopic surgery in nonhydrocephalous third ventricular colloid cysts: a feasibility study. World Neurosurg.

[2] Paniraj GL, Panigrahi M, Reddy AK (2014). Suprasellar colloid cyst: an unusual location. World Neurosurg.

[3] Fraisse T, Sirvain S (2010). Colloid cyst of third ventricle. Eur Geriatr Med.

[4] Algin O, Ozmen E, Arslan H (2013). Radiologic manifestations of colloid cysts: a pictorial essay. Can Assoc Radiol J.

[5] Swadron SP (2010). Pitfalls in the management of headache in the emergency department. Emerg Med Clin North Am.

[6] Bender B, Honegger JB, Beschorner R (2013). MR imaging findings in colloid cysts of the sellar region: comparison with colloid cysts of the third ventricle and Rathke’s cleft cysts. Acad Radiol.

